# Supplementary material for: Selective expression of variant surface antigens enables Plasmodium falciparum to evade immune clearance in vivo
Source: Nat Commun. 2022 Jul 13;13:4067. doi: 10.1038/s41467-022-31741-2 (PMC9279368; doi:10.1038/s41467-022-31741-2)
Supplement: Supplementary file 1 — Supplementary information [file 41467_2022_31741_MOESM1_ESM.pdf]

Supplementary Information

Selective Expression of Variant Surface Antigens Enables *Plasmodium falciparum* to Evade Immune Clearance *in vivo*

Marvin Chew[1,2,6], Weijian Ye[1,2,6], Radoslaw Igor Omelianczyk[1], Charisse Flerida Pasaje[3], Regina Hoo[1], Qingfeng Chen[4], Jacquin C. Niles[3], Jianzhu Chen[2,5*] and Peter Preiser[1,2,*]

[1] School of Biological Sciences, Nanyang Technological University, Singapore. [2] Singapore-MIT Alliance for Research and Technology, Antimicrobial Resistance Interdisciplinary Research Group, Singapore. [3] Department of Biological Engineering, Massachusetts Institute of Technology, Cambridge, MA, USA. [4] Humanized Mouse Unit, Institute of Molecular and Cell Biology, Agency of Science, Technology and Research, Singapore. [5] Koch Institute for Integrative Cancer Research and Department of Biology, Massachusetts Institute of Technology, Cambridge, MA, USA. [6]These authors contributed equally: Marvin Chew, Weijian Ye. *These authors jointly supervised this work: Jianzhu Chen, Peter Preiser. email: jchen@mit.edu; PRPreiser@ntu.edu.sg

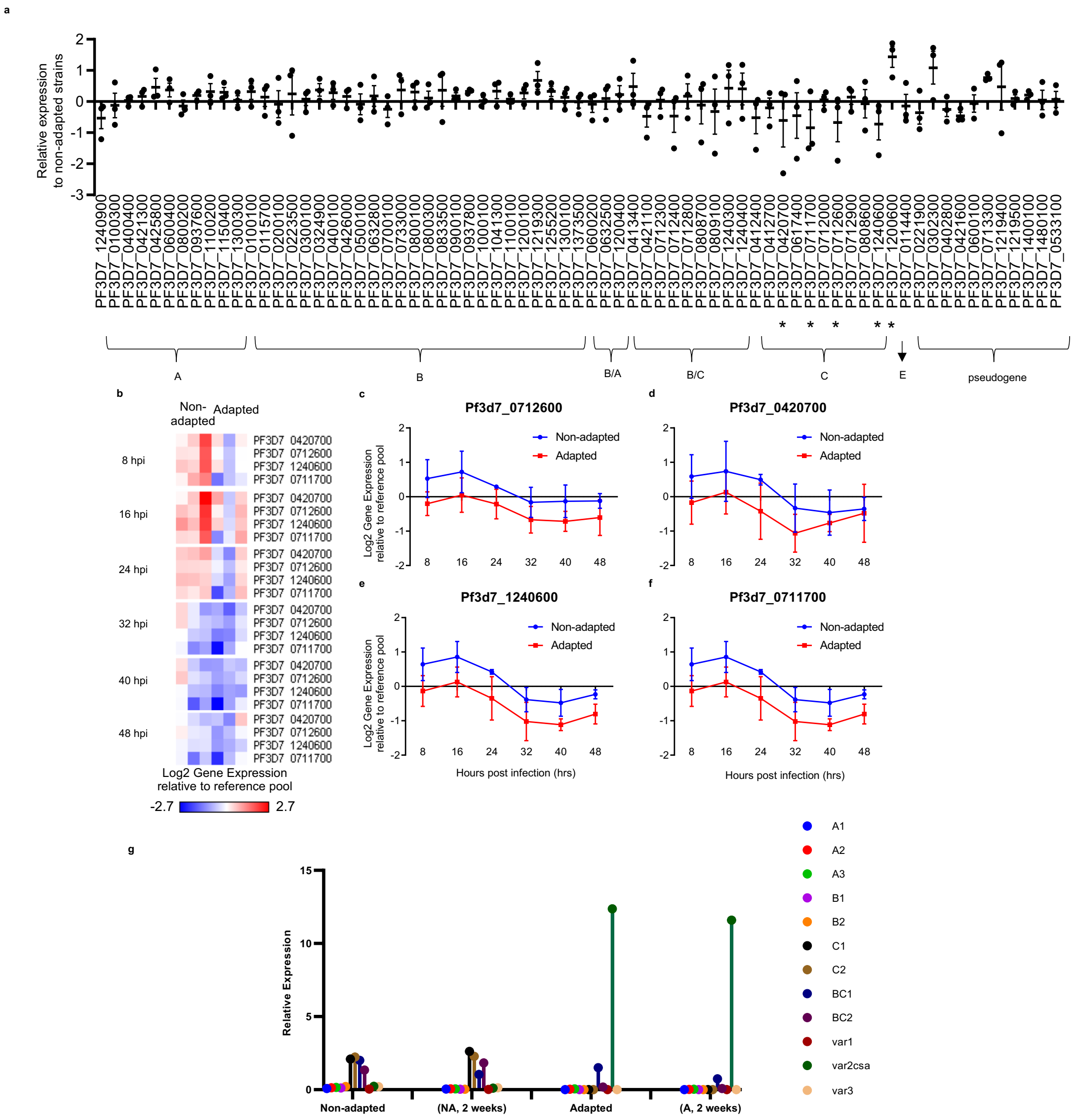

Supplementary Figure 1: Differential expression of *var* genes between adapted and non-adapted parasites. a Relative expression of individual *var* genes in adapted parasites compared to non-adapted parasites. *Var* genes are grouped according to the classification proposed by Lavstsen *et al* (Lavstsen et al., 2003). Each point represents a different pair of adapted and non-adapted parasites. *: differentially expressed gene. **b** Heatmap of the downregulated *var* genes. Each column represents a different strain of parasite, while each row represents a different *var* gene. Each cluster represent a different time point of the IDC. **c-f** Temporal gene expression profiles of the indicated *var* gene across the IDC. Each point represents the mean ± SD of n=3 biologically independent samples. **g** Quantitative RT-PCR analysis of var genes in non-adapted 3D7 and adapted 3D7 parasite from huRBC-NSG mice

and subsequent *in vitro* culture. RNA was isolated from non-adapted parasites and huRBC-NSG mouse adapted parasites directly from infected mice. Part of the adapted parasites were also cultured in static *in vitro* culture for two weeks before RNA isolation. RNA was used for quantitative RT-PCR analysis of the *var* gene family members (Group A1-3, B1-2, C1-2, BC1-2, *var1*, *var2csa* and *var3*). Source data are provided as a Source Data file.

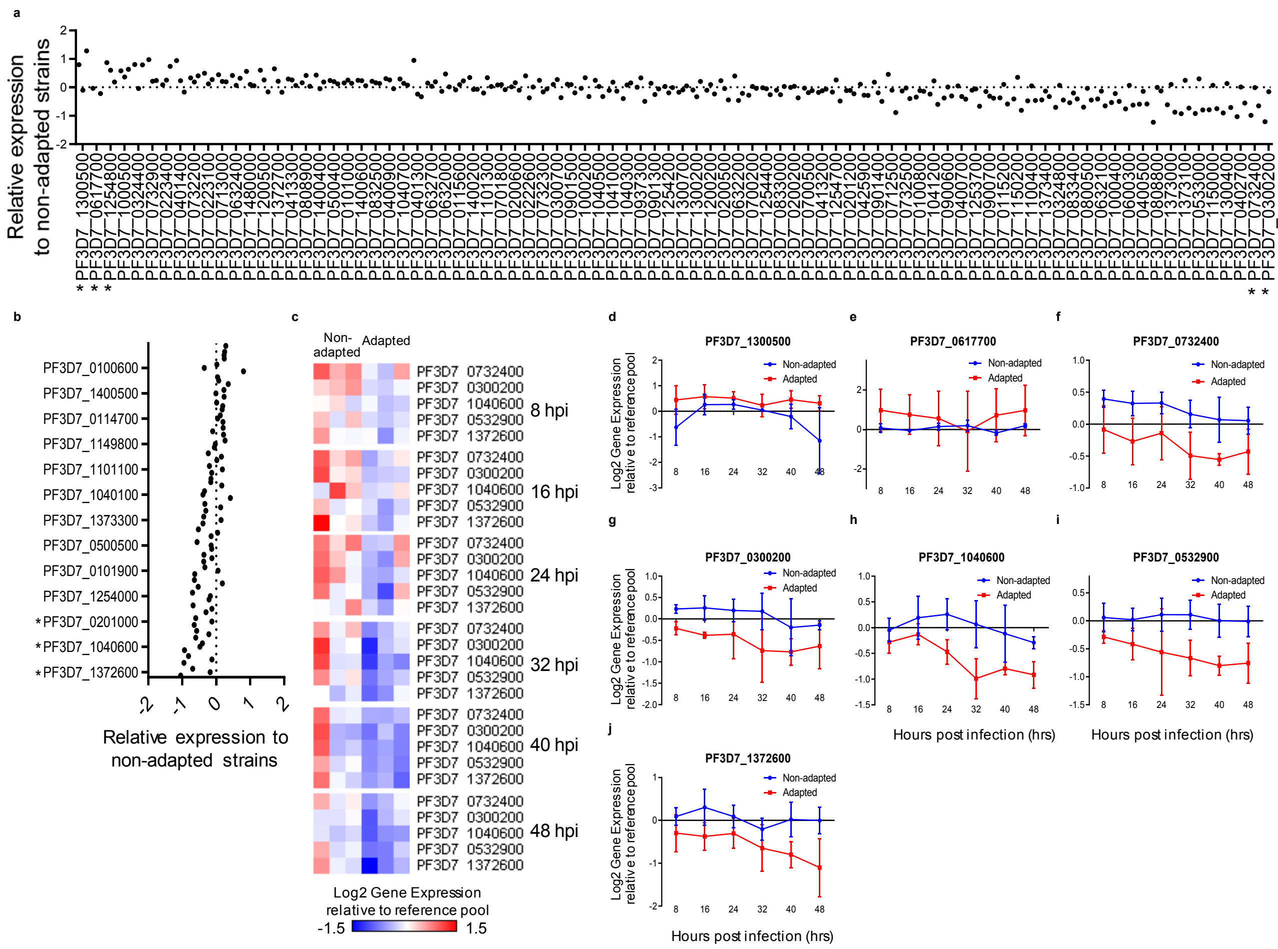

Supplementary Figure 2: Differential expression of *rif* genes between adapted and non-adapted parasites. a-b Relative expression of type A **(a)** and type B **(b)** *rif* genes in adapted parasites compared to non-adapted parasites. Each point represents a different pair of adapted and non-adapted parasites. *: differentially expressed gene. **c** Heatmap of downregulated *rif* genes. Each column represents a different strain of parasite, while each row represents a different *rif* gene. Each cluster represent a different time point of the IDC. **d-j** Temporal gene expression profiles of the indicated *rif* gene across the IDC. Each point represents the mean ± SD of n=3 biologically independent samples. Source data are provided as a Source Data file.

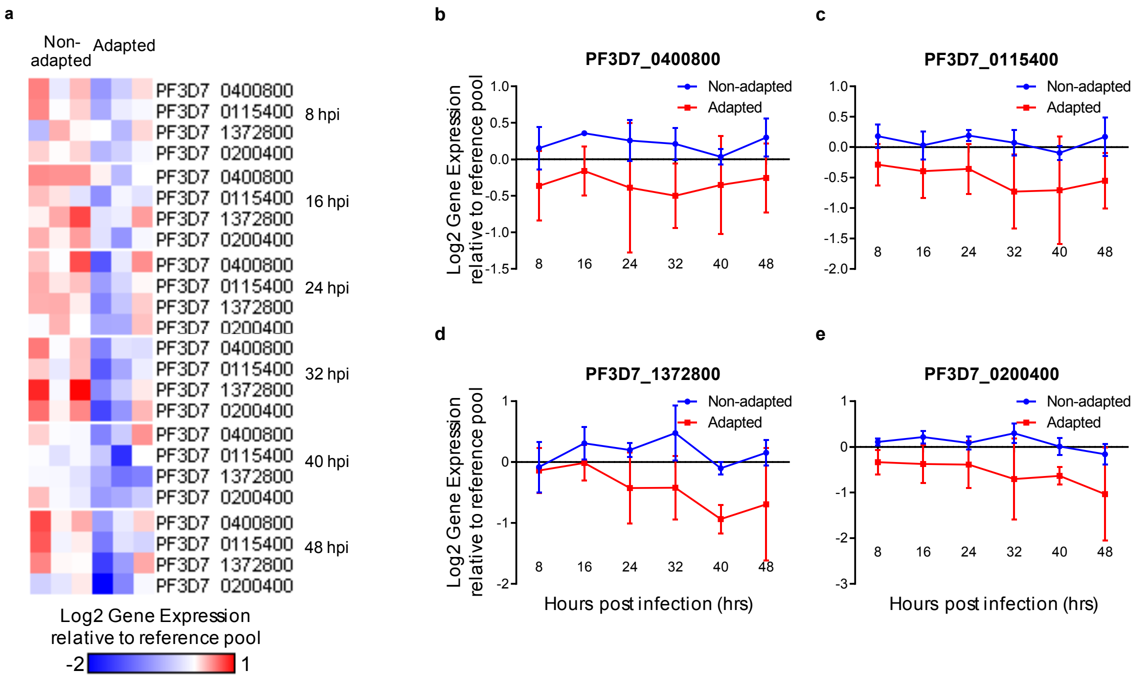

Supplementary Figure 3. Differentially expressed *stevor* genes between adapted and non-adapted parasites. a Heatmap of downregulated *stevor* genes. Each column represents a different strain of parasite, while each row represents a different *rif* gene. Each cluster represent a different time point of the IDC. **b-e** Temporal gene expression profiles of the indicated gene across the IDC. Each point represents the mean ± SD of n=3 biologically independent samples. Source data are provided as a Source Data file.

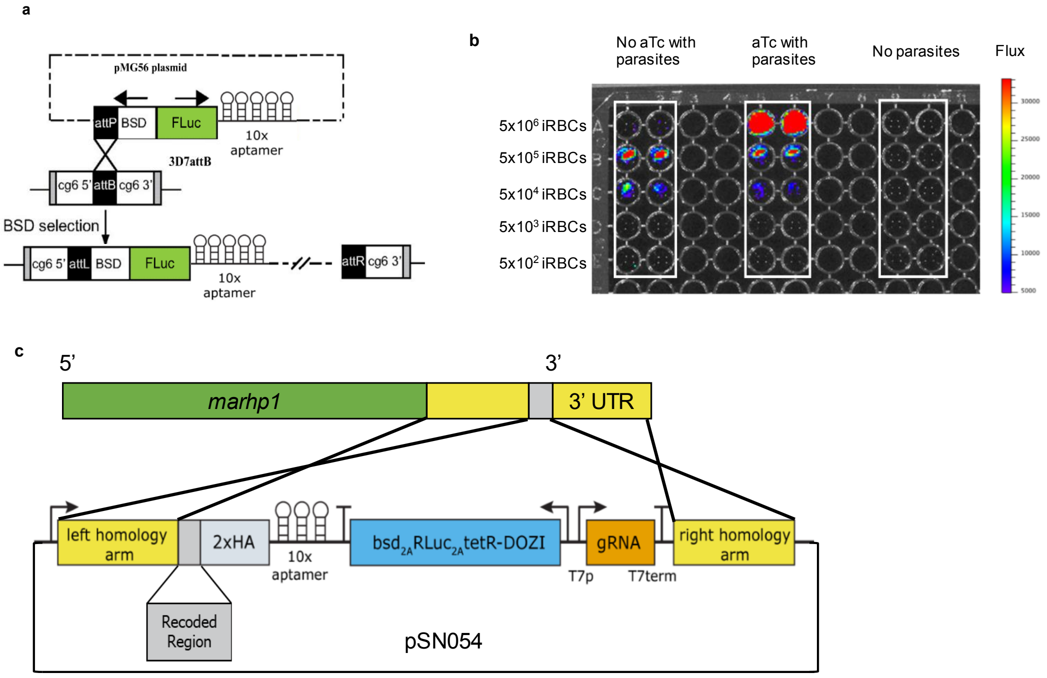

Supplementary Figure 4. Schematics of plasmids used. a Schematic of pMG56 plasmid carrying the firefly luciferase (FLuc) gene under the translational control of TetR-DOZI RNA aptamer system as previously described (Ganesan et al., 2016). AttP containing pMG56 was integrated into attB containing 3D7attB AS *P. falciparum* parasites at the dispensable cg6 gene locus to generate 3D7attB AS pMG56 parasites. **b** 3D7attB AS pMG56 parasites were serially diluted in a 96 well plate with luciferin and either in the presence or absence of aTc. Bioluminescence was analyzed using IVIS. **c** Schematic of plasmid pSN054 used to introduce a 2xHA tag as well as the 10x aptamer array to the 3' end of the *mahrp1* gene as well as components for translation regulation by the TetR-DOZI system.

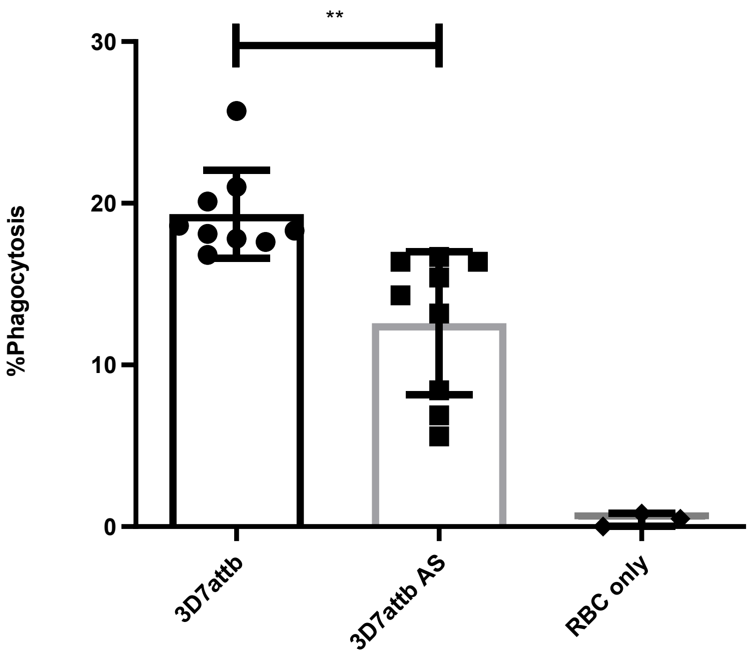

Supplementary Figure 5. Murine RAW 264.7 phagocytosis assay of adapted and non-adapted parasites. Non-adapted parasites and adapted parasites were co-cultured with murine RAW 264.7 to determine the difference in phagocytosis uptake. Data shown are mean ± SEM from n=9 replicates from 3 biologically independent experiments. Two-tailed Unpaired T-test, ** p=0.0013. Source data are provided as a Source Data file.

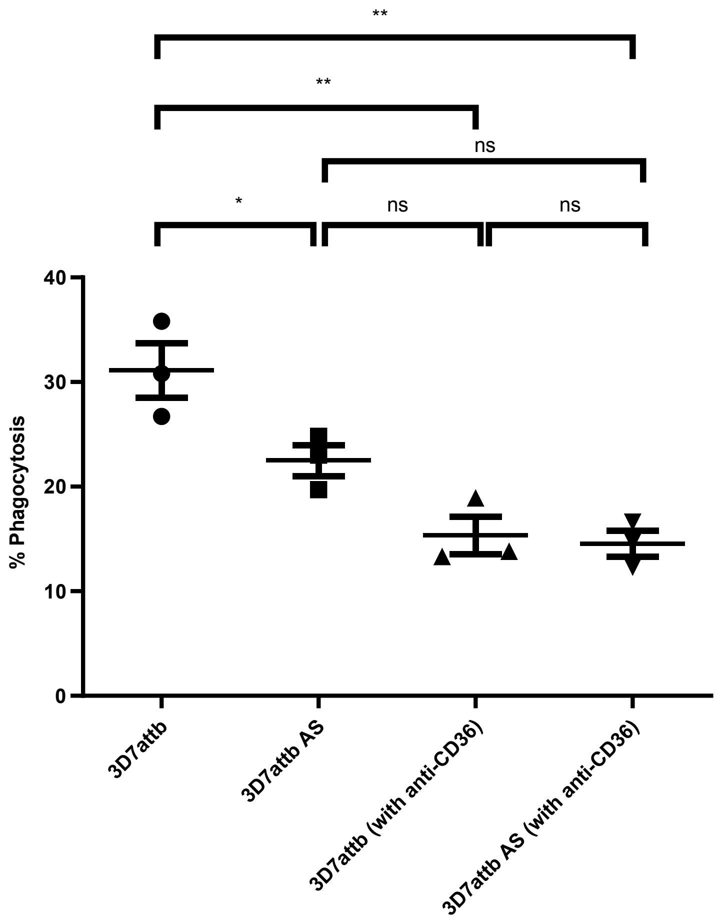

Supplementary Figure 6. Phagocytosis of iRBCs by human monocyte-derived macrophages in the presence of anti-CD36 antibody. Non-adapted and adapted 3D7attb were used to infected RBCs. The iRBCs were co-cultured with human monocyte-derived macrophages with or without anti-CD36 blocking antibody. Phagocytosis of iRBCs was determined and shown as mean ± SEM, n=3 biologically independent samples. One-way ANOVA with Tukey's multiple comparisons, *p=0.046, **p<0.002. Source data are provided as a Source Data file.

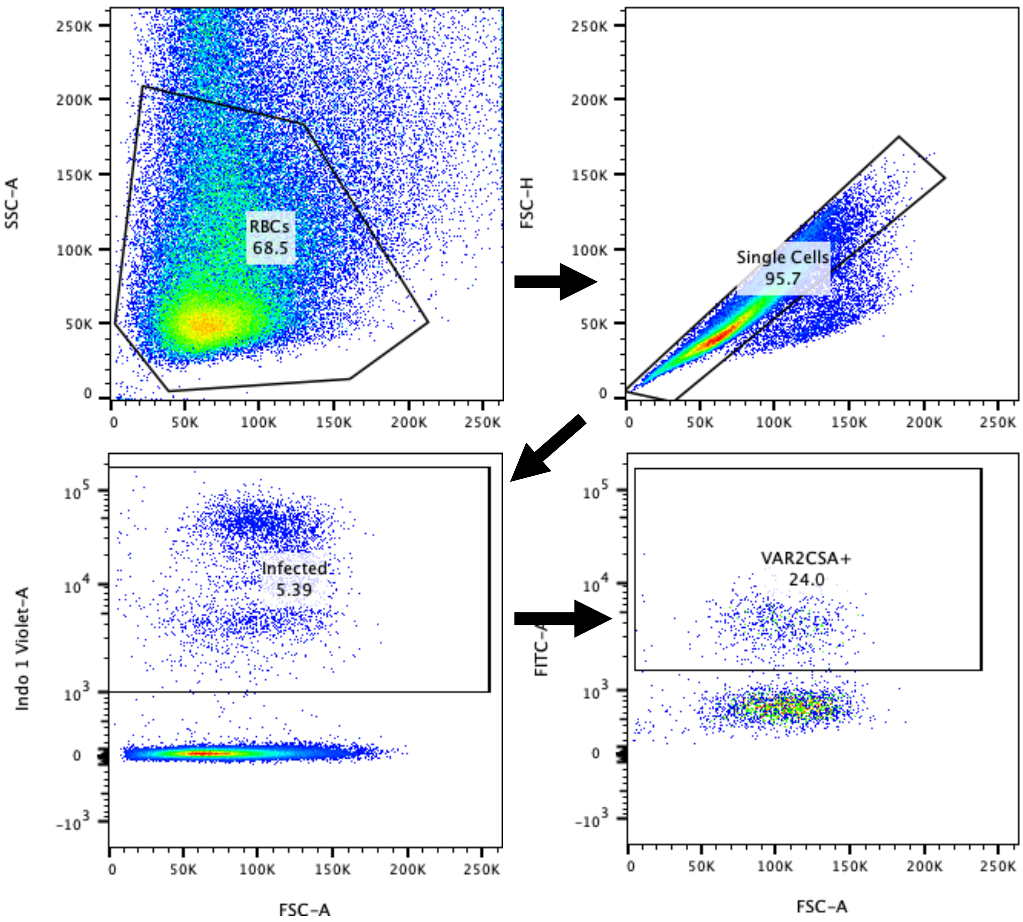

Supplementary Figure 7. Flow cytometry gating strategy for antibody PAM1.4 iRBC staining. Gating strategy used in Figure 2e to demonstrate the staining of surface VAR2CSA using antibody PAM1.4.

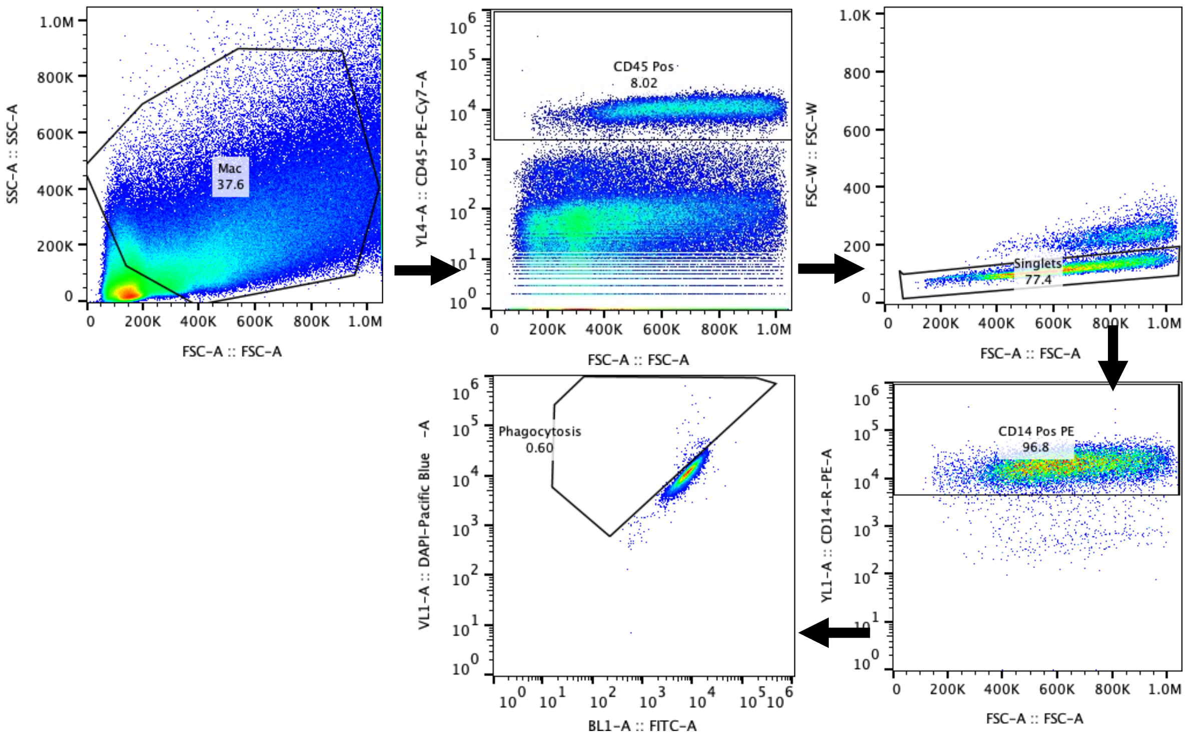

Supplementary Figure 8. Flow cytometry gating strategy for macrophage phagocytosis of iRBCs. Gating strategy used in Figure 4a, 5c, Supplementary Figure 5 & 6 to determine the phagocytosis of iRBCs.

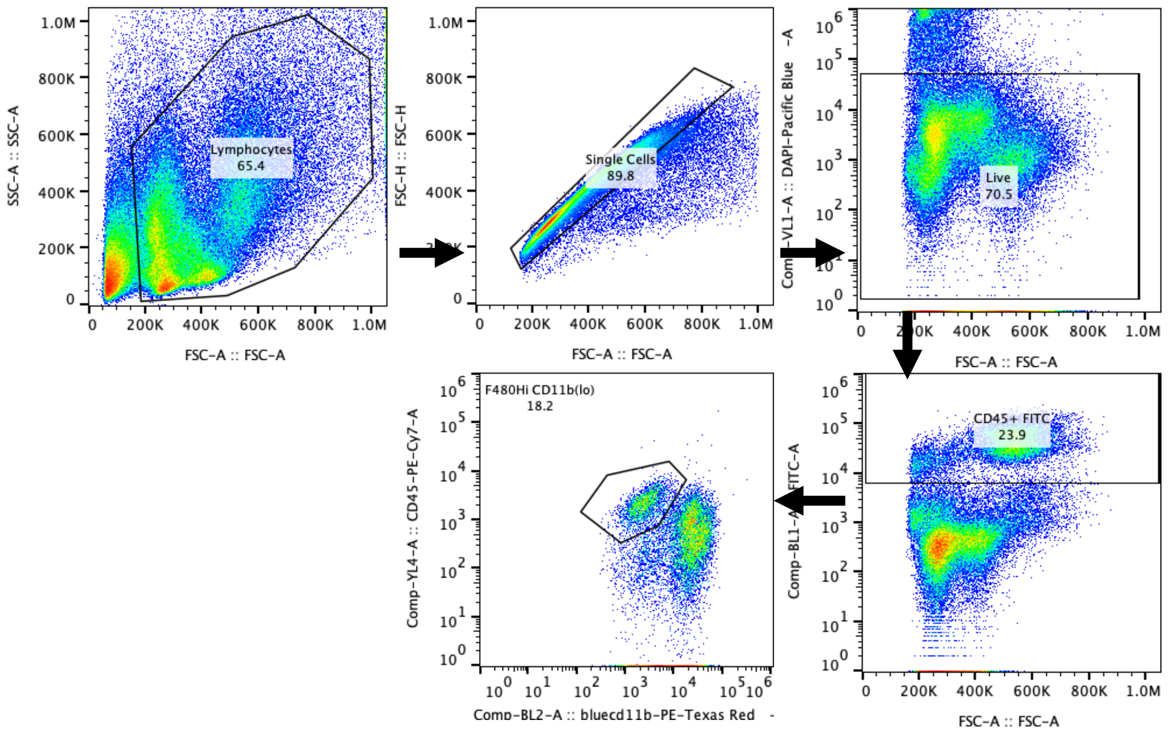

Supplementary Figure 9. Flow cytometry gating strategy for staining tissue resident macrophage in mouse spleen. Gating strategy used in Figure 4f to determine the tissue resident macrophage population in mouse spleen that are F4/80 high and CD11b low.

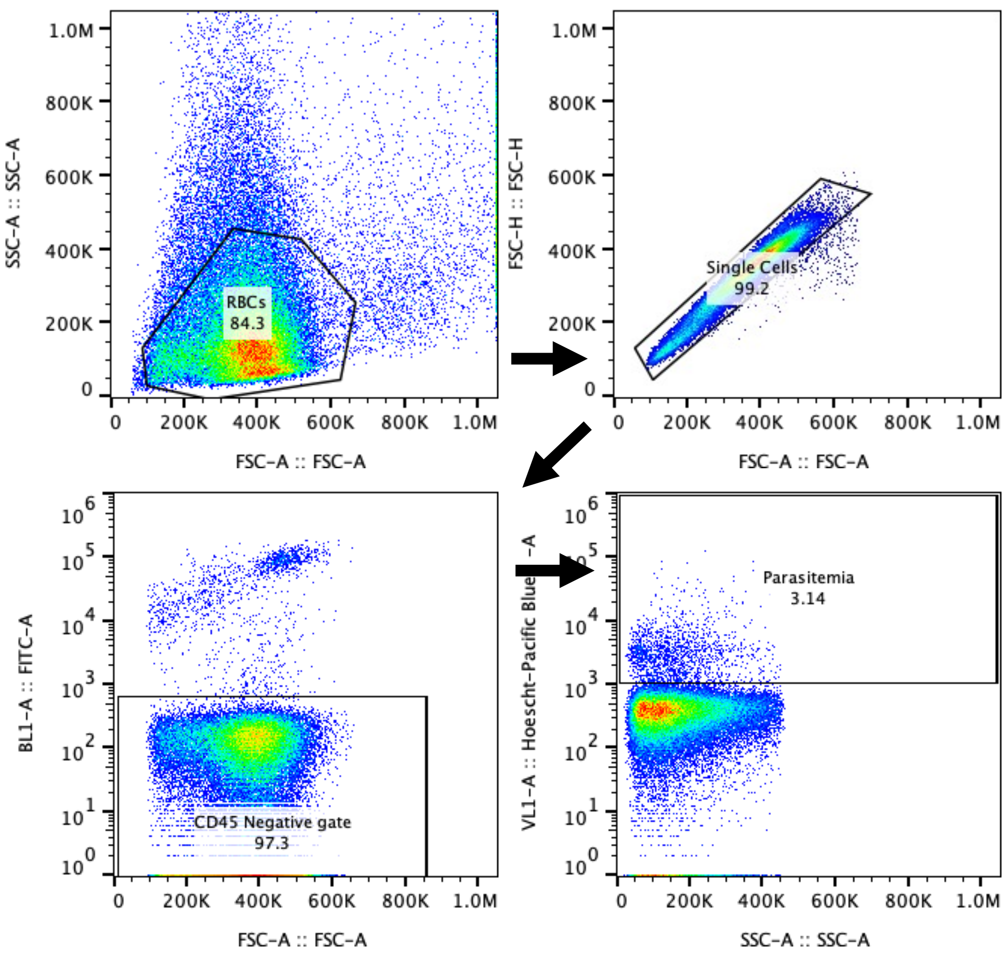

Supplementary Figure 10. Flow cytometry gating strategy for determining parasitemia for an NK cell co-culture with iRBCs. Gating strategy used in Figure 5a and 5b to determine the NK cell control of iRBCs by determining the iRBC parasitemia using CD45 to gate out the NK cell from the RBC population and DNA dye Hoechst 33342 to stain for iRBCs.

Supplementary Table 1. List of qPCR primers

Primers for *pfemp1* group A, B, C and *var2csa* were validated and referenced from Rottmann *et al*.

Gene	Primer sequence Forward (5'-3')	Primer sequence Reverse (5'-3')
A1	TTGGGRAATBTGTTAGTTAYRGCAA	CTGCAAAACTKCGWGCAAG
A2	AACCCATCTGTRRATGATATACCTATGGA	GTTCCAASGATCCATTRGATGTATTA
A3	AGGTAATGTTTTAGATGATGGTAT	ACCAGAATATACATTATTTGATACATA
B1	CATCCGCCATGCAAGTATAA	CGTGCACGATTTCGATTTTT
B2	ATCAAGGTAATTTCATACATATGTGATA	GTCCGTGCACGATTTCGATTTT
C1	CACATCGATTACATTTTAGCGTTT	TGTGGTAATATCATGTAATGG
C2	GTAGCGACAACCACGRYATCATGG	CATTGTTAACATAGTCTACCATTA
BC1	GACAAAACTTTCACCCAATAGA	AATGATCGGTGTAACCACTATC
BC2	CATCTGTTGCAAATTTATTCCAAATAC	TCAGTAGTATCAGACATAAATGCATA
var1utr	TGGCACATCTTTGGTATAAAA	AAACCTTTATATTCCTGTAAAATTCA
var2utr	CACGACATTAACAATACATGCAGA	CATTGCATTCACAGACATTGG
var3utr	CGTAAAACATGGTGGGATGA	GGCCCATTCAGTTAACCATC
